# Epilepsy, with a Peculiar Kind of Parasitic Animals in the Alimentary Canal

**Published:** 1860-11

**Authors:** G. W. Powell

**Affiliations:** Childersburg, Alabama


ATLANTA

Medical and Surgical Journal.

Vol. VI.] NOVEMBER, 1860. [No. 3.

### ORIGINAL COMMUNICATIONS.

### ARTICLE I.

*Epilepsy, with a peculiar kind of Parasitic Animals in the Alimentary Canal, reported to the Editor.* By G. W. Powell, M. D., of Childersburg, Ala.

Dear Sir—Inclosed you will find a number of Parasitic Animals, which I send you for your investigation, and with them will send you a partial history of the patient from whose stomach they have been ejected.

The patient is a young man about 22 years of age, and a subject of Epilepsy. It has been just two years since he was first attacked. The first symptoms were a spasmodic contraction of the facial muscles, and those of the neck, twisting or turning the head to one side, &c., but with no other unpleasant sensation; in fact, they were so trivial to him, that he did not mention it to any person, until about one month after, at which time he had a severe epileptic convulsion, which he has been subject to at longer or shorter intervals, until the present time. He is considered a perfect gormandizer. It was several months before he received any medical treatment, and what he has received, has been ineffectual. I had him under my treatment during September and October, 1859; and when he came to my office, he was having convulsions every two weeks—he, however, had but one under my treatment—he then left my office and went to his boarding-house, when he was attacked with symptoms of bilious fever, and that it was, I have no doubt, as we are living in a malarious district. The case, during this attack of fever, was committed to other hands, and it is only known that he was very much reduced, and remained for some time in an anæmic and anasarcous condition, having and indulging the same morbid appetite for food. No convulsions came on while in this prostrated condition; but with the return of color to the face, came again the convulsions with their usual force.

Some two months since, and after eating an unreasonable amount of food, he was attacked with nausea and vomiting, which continued until he threw up a number of Parasitic Animals, one of which was sent to me, and which I now send to you, in the blue paper.* Some of them were much larger than this one, and some of them were smaller. At the same time, he threw up a number of black insects, resembling those I send you in the white paper, and are, perhaps, the very same kind.

The premonitory symptoms of the convulsions now, are, nausea and vomiting, and the expulsion of a number of Parasitic Animals, of the two kinds I send you. The black insects that I send were ejected a few days since, and sent to

*We have had the animals under the microscope, and find one to have the shape and general character of a worm, in some respects resembling that usually found in the human bowels. It is about two lines in length and half a line in breadth, and of a pale red color. The other is an insect unlike anything usually found in the canal. It is from three-fourths of a line to a line in length, and from a fourth to half a line thick, of a dark reddish color—has six legs and two autennæ, and in appearance intermediate between the beetle and flea.—[EDITOR.

me in a letter—the letter I will send to you, and will refer you to the writer for other facts in this case. I see from his letter, that he intended to send some that were passed off by the bowels, but he failed to do it. He informed me the other day, in a personal interview, that those black insects could move with nearly the agility of a flea, and that it was with difficulty that they could be caught on the floor. I have also been informed, that this worm lived four days in a watch crystal, with nothing else in it. These parasites have been presented to several physicians, and they all plead ignorance of the knowledge of their existence in the human subject heretofore. I send you the parasites, hoping you will examine them microscopically and pathologically, and give me some information in regard to their genera.

Perhaps you may think it proper for me to give my opinion in the case, as they were sent me for investigation, which would be imperfect if I did. I have searched all my pathology, and other works that treat of parasites, and I can find nothing that will bear out the case before us. In all probability, this case has not, never did have, and may never have a parallel; as far as my knowledge goes, no writer on pathology describes such animals.

My opinion, however, is, that the parasites are not the cause of the epilepsy; but if they are, they have existed in the system at, and previous to the first attack. I am rather of the opinion, that they did not exist until after the anœmic condition of the system above spoken of; and that, if they did exist before, in all probability, it was in the form of the black insects, and that it may have required the 22 months for them to attain the size and appearance of the worms that have been ejected from the stomach; and that it was from the irritation they produced, that brought on the convulsions.

Some pathologists have contended, that parasitic animals are the result of spontaneous or equivocal generation, springing into existence without parents, though some action going on within the system. It is said, "that parasitic animals of the human frame, have no prototype in nature;" but, in the first place, the fact, that no such prototypes have been discovered, is no proof of their non-existence; and, secondly, animals of the same species as the parasite, may be constantly before us in the outer world, but with characters so different that they cannot be recognised. It may be, therefore, that of the innumerable germs which fill the external world, some may find admittance into our frames, and there expand into beings apparently different from anything around us.

